# A Current Review of Targeted Therapeutics for Ovarian Cancer

**DOI:** 10.1155/2010/149362

**Published:** 2010-01-03

**Authors:** Susana M. Campos, Sue Ghosh

**Affiliations:** ^1^Dana Farber Cancer Institute, Harvard Medical School, Boston, MA 02115, USA; ^2^Brigham and Women's Hospital, Harvard Medical School, Boston, MA 02115, USA

## Abstract

Difficult to detect, ovarian cancer typically presents at an advanced stage. Significant progress has been achieved in the treatment of ovarian cancer with therapeutics focused on DNA replication or cell division. However, despite sensitivity to induction chemotherapy the majority of patients will develop recurrent disease. Conventional agents for recurrent disease offer little in terms of long-term responses. Various targeted therapeutics have been explored in the management of ovarian cancer. These include monoclonal antibodies to epidermal growth factor receptors, small molecule tyrosine kinase inhibitors, monoclonal antibodies directed at the vascular endothelial growth factor (bevacizumab), and the small tyrosine kinase inhibitors that target the vascular endothelial growth factor receptor. Recently, several other agents have come forth as potential therapeutic agents in the management of ovarian cancer. These include monoclonal antibodies to the folate receptor, triple angiokinase inhibitors, PARP inhibitors, aurora kinase inhibitors, inhibitors of the Hedgehog pathway, folate receptor antagonists, and MTOR inhibitors.

## 1. Introduction

Various targeted therapeutics have been explored in the management of ovarian cancer. These include monoclonal antibodies to Her 2 neu [1, 2] and other epidermal growth factor receptors [3] (i.e., Trastuzumab [1], Pertuzumab [2], and EMD 7200 [3]), small molecule tyrosine kinase inhibitors that targeted the various EGFR receptors (gefitinib [4], erlotinib [5], CI-1033 [6]), monoclonal antibodies directed at the vascular endothelial growth factor [7–19] (bevacizumab), and the small tyrosine kinase inhibitors that target the vascular endothelial growth factor receptor [20–25]. Recently, several other agents have come forth as potential therapeutic agents in the management of ovarian cancer. These include monoclonal antibodies to the folate receptor, triple angiokinase inhibitors, PARP inhibitors, aurora kinase inhibitors, inhibitors of the Hedgehog pathway, folate receptor antagonists, and MTOR inhibitors.

This paper will explore the current data on the various targeted approaches in ovarian cancer. Attention will be directed at understanding the molecular mechanisms of these agents balanced with their application to clinical practice.

## 2. Angiogenesis

Enthusiasm for cytotoxic agents in the management of ovarian cancer has been tempered by the emergence of resistance. As such, a focus on alternative innovative therapeutics has emerged. One such direction is the inhibition of angiogenesis. Angiogenesis is one of the cardinal processes leading to invasion and metastasis of solid tumors. The angiogenic-signaling pathway may be triggered by the release of angiogenic ligands such as the vascular endothelial growth factor from tumor cells. Tumor angiogenesis is well established as essential for the growth and metastasis of solid tumors, [26–28] This process involves the recruitment of mature vasculature and circulating endothelial cells [29, 30] and proangiogenic soluble mediators one of which includes the vascular endothelial growth factor (VEGF) [31]. This factor has several known activities [31], such as mitogenesis, angiogenesis, endothelial survival, enhancement of vascular permeability, and effects on hemodynamic status. In ovarian cancer increased levels of VEGF are associated with poor prognosis and have been confirmed in multivariate analysis as an independent prognostic indicator of survival [28, 32–38]. Given the poor long-term responses appreciated with conventional cytotoxic agents that target VEGF have taken center stage. 

Agents targeting angiogenesis include monoclonal antibodies to the VEGF ligand [7–19], small tyrosine kinase inhibitors that target the vascular endothelial growth factor receptor [20–25], and soluble decoy VEGF receptors [39, 40]. The most studied agent to date has been bevacizumab, a recombinant humanized monoclonal antibody to the VEGF ligand. 

To date several investigators [7–19] (Table 1) have explored bevacizumab as a single agent or in combination with chemotherapy in the management of advanced ovarian cancer. 

Several studies in both the upfront and in the recurrent setting are underway. GOG 218 is a randomized placebo controlled three-arm study examining the role of bevacizumab in combination with carboplatin and paclitaxel and also as a maintenance therapy. ICON-7 is a two arm trial comparing carboplatin and paclitaxel (six cycles) versus carboplatin, paclitaxel, and bevacizumab (7.5 mg/kg) for six cycles followed by 12 cycles of maintenance bevacizumab.Campos et al. [20] is conducting a phase II trial of carboplatin/paclitaxel/bevacizumab in optimally and suboptimally debulked patients. Patients achieving a clinical complete response, partial response, or stable disease are subsequently randomized to either bevacizumab for 12 months or the combination of bevacizumab and erlotinib. Preliminary safety results have noted an increase in hypertension but to date no evidence of gastrointestinal perforations.

Given the recent data that has emerged on the role on intraperitoneal chemotherapy [41–43] investigators are exploring the role of IP chemotherapy with IV bevacizumab. Several abstracts were highlighted at the recent American Society of Clinical Oncology meeting. Konner et al. [44] and McKeekin et al. [45] in independent studies reported the feasibility of utilizing bevacizumab (IV) in conjunction with intraperitoneal therapy. One bowel perforation [44] was noted the Konner study while McKeekin et al. [45] colleagues noted one deep venous thrombosis and one fistula.

**Figure 1:**
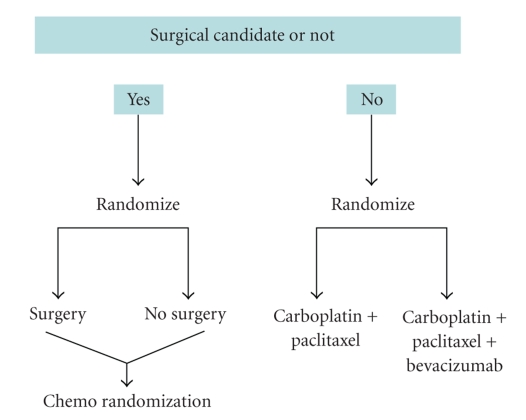
GOG 213.

In the recurrent setting several trials are being conducted. The OCEANS trial is a randomized study of carboplatin/gemcitabine and bevacizumab (NCT 00434642) versus carboplatin/gemcitabine. GOG 213 (Figure 1) is randomized trial in recurrent ovarian cancer patients. Patients are stratified as to whether or not they are surgical candidates. If the patients are deemed to be surgical candidates they are randomized to surgery or no surgery followed by randomization to chemotherapy. If patients are randomized to no surgery they are subsequently randomized to carboplatin and paclitaxel or carboplatin/paclitaxel and bevacizumab.

The combination of carboplatin/DOXIL and bevacizumab is also being studied. The later trial may prove to be intriguing given the recently reported results of the CALYPSO trial [46]. In the CALYPSO trial the combination of Carboplatin-Doxil demonstrated a superior therapeutic index (benefit/risk ratio) versus current standard, carboplatin-paclitaxel.

## 3. Small Molecules that Target the VEGFR Receptor

**Figure 2:**
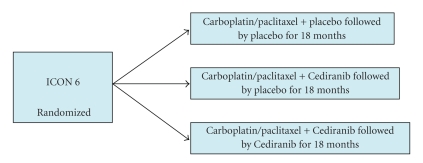
ICON-6.

Small molecule tyrosine kinase inhibitors that target the vascular endothelial growth factor receptor are currently being investigated in numerous clinical trials. AZD2171 (Cediranib) is a novel oral tyrosine kinase inhibitor of VEGFR2, VEGFR1, and c-kit. Matulonis et al. [21] reported the initial results of this agent in the management of patients with recurrent ovarian cancer. Five patients had confirmed partial responses with an overall response rate of 18.5%. Three patients had stable disease lasting 30, 27+, and 24 weeks. Hirte et al. [22] reported a response rate of 40.5% in platinum sensitive patients and a response rate of 29% in platinum resistance disease with AZD 2171 (Cediranib). Prevalent side effects included fatigue and hypertension. Currently, ICON-6 is conducting a study of AZD2171 (Cediranib) in platinum sensitive relapsed ovarian cancer in a three arm randomized placebo-controlled phase III trial in combination with paclitaxel and carboplatin. (Figure 2).

Pazopanib is tyrosine kinase inhibitor of vascular endothelial growth factor receptor (VEGFR) −1, −2, and −3, platelet-derived growth factor receptor (PDGFR) −*α* and −*β*, and c-Kit. Friedlander et al. [24] have reported activity with pazopanib in women with advanced epithelial ovarian cancer. Eleven of 36 subjects (31%) experienced a cancer antigen-125 (CA-125) response to pazopanib. Overall response rate based on modified Gynecologic Cancer Intergroup (GCIG) criteria (incorporating CA-125, Response Evaluation Criteria in Solid Tumors (RECIST), and clinical assessment) was 18% in subjects with measurable disease at baseline and was 21% in subjects without measurable disease at baseline. Median PFS was 84 days.

Sunitinib, an inhibitor that targets the VEGFR 1, 2, 3, and platelet–derived growth factor receptors, has also been studied in the management of patients with recurrent ovarian cancer. Biagi et al. [25] investigated the role of sunitinib in the management of patients with recurrent ovarian cancer. Sunitinib was administered at 50 mg every day on a 4-week on 2-week off schedule. Noted in this study was the development of pleural effusions during the 2-week rest period. Of the seventeen patients that were studied 12% of patients had a partial response, and 59% of patients had disease stabilization. Currently the Harvard Cancer Center Gynecological Group (NCT00768144) is conducting a phase II trial using sunitinib in refractory ovarian caner patients. The dose of sunitinib is held constant at 37.5 mg every day. 

AMG 706 is an investigational inhibitor of vascular endothelial growth factor receptors 1, 2, and 3, platelet-derived growth factor receptor, and stem-cell factor receptor. A Phase II Evaluation of AMG706 (NCT00574951) in the Treatment of Persistent or Recurrent Epithelial Ovarian Fallopian Tube or Primary Peritoneal Cancer is currently active.

Matei et al. [26] reported on the activity of sorafenib in patient with recurrent ovarian cancer. Sorafenib is a tyrosine kinase inhibitor targeting raf and other receptor kinases (VEGF-R, PDGF-R, Flt3, c-KIT). Patients received sorafenib at 400 mg QD. Patients in this study have a 3% partial response, and 20% of patients had stable disease for > than 6 months. Toxicities included rash, metabolic abnormalities, gastrointestinal, cardiovascular, and pulmonary toxicity.

## 4. VEGF Trap (Aflibercept)

VEGF trap (Aflibercept) is fusion protein containing the VEGF binding regions of both VEGFR-1 and 2 linked through the Fc region of a human IgG1. Aflibercept binds VEGF-A and neutralizes all VEGF-A isoforms plus placental growth factor. This agent is currently being explored in platinum resistant ovarian cancer. Columbo et al. [39] reported the results of VEGF Trap in patients with symptomatic malignant ascites. Aflibercept, 4 mg/kg, i.v. was administered every 2 weeks, in patients with advanced ovarian cancer and symptomatic ascites requiring frequent paracentesis. Primary endpoint was repeat paracentesis response rate (RPRR) defined as at least a doubling of time to the first paracentesis compared to a baseline average. Patients received 1–13 cycles of aflibercept. The authors reported that the time to the first paracentesis was 12–205 days. Eight out of ten evaluable patients achieved a RPRR response as per protocol. Adverse events included bowel obstruction, nausea, vomiting, anorexia, edema, and 1 case of bowel perforation. Tew et al. [40] reported the preliminary results of a randomized phase II study in patients with recurrent platinum-resistant epithelial ovarian cancer. VEGF Trap was (2 or 4 mg/kg) administered intravenously every 2 weeks in patients with recurrent ovarian cancer was conducted. Five partial responses in a sample size of 45 patients (11%) were reported.

## 5. Epidermal Growth Factor Inhibitors

In addition to the VEGF inhibitors, the epidermal growth factor receptor (EGFR) has emerged as an attractive target [47–49]. The activation of EGFR signaling pathways is known to increase proliferation, angiogenesis, and decrease apoptosis. Several strategies that target the EGFR in gynecologic cancers have included monoclonal antibodies [1–3], (trastuzumab, pertuzumab, EMD7200) and tyrosine kinases inhibitors [4–6] (gefitinib, erlotinib, lapatinib and CI-1033). Bookman and colleagues [1, 50] reported a response rate of 7% in a phase II trial of ovarian cancer patients treated with trastuzumab. Kaye et al. [51], Amler et al. [52], and Makhija et al. [53] in independent studies examined pertuzumab, a humanized recombinant monoclonal antibody that inhibits the dimerization of HER2 with EGFR, HER 3, and HER4, in patients with ovarian cancer. As a single agent there were only modest responses. Gordan et al. [54] recently published the clinical activity of pertuzumab in advanced ovarian cancer. There were five partial responses (response rate 4.3%), eight patients (6.8%) with stable disease lasting at least 6 months, and 10 patients with CA-125 reduction of at least 50%. Median progression-free survival (PFS) was 6.6 weeks. Twenty eight percent of the tumor biopsies were pHER2+ by ELISA. Of note the progression free survival for pHER2+ patients was 20.9 weeks (*n* = 8) versus 5.8 weeks for pHER2−.

Several studies are ongoing. The EORTC have recently completed a trial investigating erlotinib as maintenance therapy following first-line chemotherapy in patients with ovarian cancer (NCT00263822). A phase II open label trial of erlotinib and bevacizumab is being conducted by Alberts et al. in patients with advanced ovarian cancer (NCT00696670).

Unlike other disciplines there is lack of data in the gynecological literature on who, if any, will benefit from EGFR inhibitors. Schilder et al. [55] reported that in a sample size of 55 ovarian cancer patients 3.6% had mutations in the EGFR tyrosine kinase domain and that the mutation correlated with a response to gefitinib. Exploratory analyses in the pertuzumab studies [51–53] suggested that patients with platinum resistant disease and low levels of HER3 mRNA might benefit from pertuzumab. An additional study by Tanner et al. [56] demonstrated an influence of HER 3 expression on the survival of patients with ovarian cancer. 

Selection of ovarian cancer patients with EGFR amplifications, increased pHER2, and low expression of HER 3 ratios may represent the selected few that may respond to EGFR inhibitors.

## 6. Combination Therapy with EGFR and VEGF Inhibitors

EGFR activation has been reported to promote VEGF [57] secretion. Several clinical studies are exploring the combination of EGFR inhibitors and VEGF inhibitors. Nimeiri et al. [12] investigated the clinical activity and safety of bevacizumab and erlotinib patients with recurrent ovarian, primary peritoneal, and fallopian tube cancer. In this study patients were heavily pretreated. Two patients had a fatal bowel perforation.

Currently investigators at the Harvard Cancer Center are conducting a randomized phase II trial of Bevacizumab or Bevacizumab and Erlotinib as First Line Consolidation Chemotherapy after Carboplatin, Paclitaxel, and Bevacizumab (CTA) Induction Therapy for Newly Diagnosed Advanced Ovarian, Fallopian Tube and Primary Peritoneal Cancer & Papillary Serous Mullerian Tumors (NCT00520013) [20].

## 7. Platelet Derived Growth Factor Inhibitors

Platelet-derived growth factor (PDGF) a prototype for understanding the function of growth factors and receptor tyrosine kinases (TK) [58] induces cell growth and survival, transformation, migration, vascular permeability, and wound healing [59]. PDGF receptor (PDGFR) activation in cancer occurs as a consequence of gene amplification, chromosomal rearrangements, or activating mutations [60–62]. PDGFR activation is critical to tumor initiation in addition to functioning as a mediator of connective tissue stroma [63].

PDGFR has been shown in 50–80% of ovarian tumors [63]. Several agents that target the PDGFR have been studied. These include imatinib mesylate [63–66], sorafenib, [17, 26], sunitinib [25], dasatinib [67], 3G3 [68], and CDP 860 [69]. Imatinib mesylate is a selective Abl, c-Kit, and PDGFR inhibitor. Three phase II clinical trials [64, 70, 71] in patients with ovarian cancer failed to demonstrate clinical benefit. 

The GOG (170 M) is currently studying dasatinib in a Phase II Evaluation of Dasatinib in the Treatment of Persistent or Recurrent Epithelial Ovarian, Fallopian Tube, or Primary Peritoneal Carcinoma.

BIBF1120 [72] is a novel agent. It is a triple angiokinase inhibitor that targets the VEGFR, PDGRF, and the fibroblast growth factor receptor (FGFR). Sustained pathway inhibition is a distinct feature of this agent. Ledermann et al. [73] recently conducted a randomized phase II placebo-controlled trial using maintenance therapy to evaluate the vascular targeting agent BIBF 1120 following treatment of relapsed ovarian cancer. The 36-week PFS rate for BIBF 1120 was 15.6% and 2.9% for placebo. The authors concluded that maintenance BIBF 1120 could delay disease progression in ovarian cancer patients who had previously responded to chemotherapy.

## 8. Folate Receptor Inhibitors

Folic acid is an essential vitamin and of importance for one-carbon transfer processes medicated by enzyme systems involved in DNA synthesis [74]. Increased expression of *α*-FR has been described in various tumor tissues, including ovarian, endometrial, and breast cancer [75]. While the function of *α*-FR in cancers is not fully understood, folates are critical metabolites for nucleotide synthesis and methylation reactions. Its overexpression might confer a tumor growth advantage by increasing folate availability to cancer cells [75]. Over 90% of nonmucinous ovarian cancers overexpress *α*-FR [76]^.^

Several strategies have been employed to target the folate receptor. Some of these include the use of anti-*α*-FR antibodies or folic acid conjugates. There has also been recent research to show that *α*-FR may have a potential as a target for immunotherapeutic approaches in ovarian cancer. *α*-FR is a tumor-associated antigen that induces detectable immune responses in 70% of patients with breast and ovarian cancer [77]. The presence of endogenous immune reactivity raises the possibility that the immune response could be further enhanced by vaccines targeting the *α*-FR. Hernando et al. [78] presented a case of a women with recurrent epithelial ovarian cancer treated with a vaccination regimen created with autologous dendritic cells engineered with mRNA-encoded *α*-FR [78]. An initial contrast-enhanced CT of the abdomen before vaccination had shown para-aortic lymph node metastasis at the level of the left renal hilus and lower abdominal aorta. Follow-up CT 16 months after last vaccination depicted a more than 50% regression of lymph node metastasis and a dramatic decrease in CA125 concentrations 4 weeks after the first vaccination [78]. 

Farletuzumab (MORAb-003) is a monoclonal antibody to *α*-FR that activates antibody-dependent cell-mediated cytotoxicity and complement-mediated toxicity [79]. In a recent Phase II trial of 54 patients [80] with platinum-sensitive relapsed disease patients who received combination therapy exhibited a prolongation of their remission when compared to their previous remission. Ongoing clinical trials looking at Farletuzumab include a Phase III trial comparing the efficacy and safety of intravenous carboplatin and taxanes with and without farletuzumab in subjects with first platinum-sensitive relapse, a Phase II trial examining intravenous paclitaxel with and without farletuzumab in patients with first platinum-resistant or refractory relapse. 

EC145 is a drug that is specifically designed to enter cancer cells via the folate vitamin receptor (FR). Early clinical evidence in a small number of phase I patients suggests that EC145 may have antitumor effect in women with advanced ovarian cancer. Current independent studies include a study of EC145 in patients with advanced ovarian and endometrial cancers (NCT00507741) and a study in patients with platinum resistant ovarian cancer with a combination of Doxil and EC145 Combination Therapy (NCT00722592).

## 9. Poly-ADP-Ribose Polymerase (PARP) Inhibitors

Between 5 and 10% of all ovarian cancer cases are associated with inheriting a mutation in the BRCA1 or BRCA2 gene [81]. The lifetime risk of ovarian cancer for BRCA1 and BRCA2 mutation carriers is estimated at 40–50% and 10–20%, respectively. BRCA1 and BRCA2 are essential for the repair of double strand DNA breaks (DSBs) and maintenance of genomic stability [82]. 

Poly (ADP-ribose) polymerase (PARP) is a key nuclear enzyme involved in the repair of DNA single-strand breaks (SSBs) using the base excision repair pathway [83]. PARP-1 and PARP-2 are the only members of the PARP family known to be activated by DNA damage, and PARP-1 has been best characterized. PARP inhibition results in the accumulation of DNA SSBs, which may lead to DSBs. Thus, the use of PARP inhibitors in BRCA mutation carriers uses the concept of synthetic lethality and hence can be described a therapeutic exploitation. 

In the first human phase I clinical trial using Olaparib (AZD2281, KU-0059436; AstraZeneca) an oral small-molecule PARP-1 inhibitor, toxicities included nausea, vomiting, anorexia, and fatigue. Efficacy has been reported. Olaparib has shown antitumor activity in BRCA-associated ovarian cancer [84, 85]. Fifty patients were treated at various doses, of which 41 were BRCA1 mutation carriers, eight were BRCA2 mutation carriers and one had a compelling family history for BRCA mutation. Of the 46 patients with evaluable disease, 41% reached either a complete or partial response. Eleven percent had meaningful stabilization of disease for 4 months, giving a total clinical benefit rate of 52%. The median response duration was 30 weeks.

Recently reported were the results of a phase II trial of the oral PARP inhibitor Olaparib (AZD 2281) in BRCA-deficient advanced ovarian cancer [86]. An international, phase II study examined two cohorts of patients that received oral olaparib in 28-day cycles, initially at the MTD, 400 mg bid (33 pts), and subsequently at 100 mg bid (24 pts). The confirmed overall response rate was 33% at 400 mg bid dose and 12.5% at 100 mg bid dose. Clinical benefit rate (ORR and/or confirmed ≥50% decline in CA125) was 57.6% at 400 mg bid and 16.7% at 100 mg bd. Toxicity was mild.

Olaparib is currently being evaluated in randomized Phase II trials in platinum-sensitive recurrent ovarian cancer, and in known BRCA or high grade recurrent ovarian cancer. It is also being compared with pegylated liposomal doxorubicin in patients with BRCA mutated ovarian cancer with a 0-12 month platinum-free interval (NCT00628251). 

Other PARP inhibitors are also being evaluated in BRCA mutation carriers with cancer, including AG0146999 (Pfizer) ABT888 (Abbott), BSI-201 (Bipar), INO-1001 (Inotek/Genentech), and MK4827 (Merck). 

The use of PARP inhibitors might be extended to sporadic ovarian cancers with homologous recombination defects. These sporadic tumors seem to phenocopy BRCA1/2 deficient tumors even though they do not possess the germline mutations in either gene. This phenomenon is called “BRCAness.” This can occur due to loss of heterozygosity, hypermethlyation, and haploinsufficiency (inactivation of one BRCA allele), thereby, genetically silencing the BRCA gene without an actual germline mutation. A recent study suggests that over 50% of high-grade serous ovarian cancer had loss of BRCA function, either by genetic or epigenetic events [87]. A randomized placebo-controlled trial of olaparib as a maintenance therapy in patients with serous (sporadic) ovarian cancer at high risk for recurrence is now underway. 

## 10. Aurora Kinase Inhibitors

Aurora kinases are protein kinases that are important mitotic regulators [88, 89]. They are central to many cellular functions notably mitosis, centromere separation, as well as mitotic spindle formation. Three aurora kinases (A, B, C) exist. The activity of aurora kinase is cell cycle dependent and active during the G2M phase of the cell cycle. Several investigators [88, 90, 91] have described the oncogenic potential of these proteins. Aurora-A also phosphorylates the tumor suppressor protein p53, resulting cell cycle progression [92].

Aurora A is overexpressed in 83% of human epithelial ovarian carcinomas [93]. In addition, amplification of human chromosome 20q13.2, which contains Aurora-A, frequently occurs in ovarian cancer [94]. Aurora kinase A has been significantly associated with tumor grade, FIGO stage, and survival [93, 95].

Lin et al. [96] studied the role of MK-0457, a small molecule pan-aurora kinase inhibitor in ovarian cancer cell models. Two chemosensitive human ovarian cancer cell lines, HeyA8 and SKOV3ip1, were used to study the effects of aurora kinase inhibition. Additionally two chemoresistant cell lines (Hey A8-MDR and A2780-CP-20) were also studied. Both cell lines showed that aurora kinase inhibition alone significantly reduced tumor burden. Combination treatment with docetaxel resulted in significantly improved reduction in tumor growth beyond that afforded by docetaxel alone (*P* < or = .03). Scharer et al. [97] also reported that aurora kinase inhibitors synergize with paclitaxel to induce apoptosis in ovarian cancer cells.

Manfredl et al. [98] reported the antitumor activity of MLN8054, an orally active small molecule inhibitor of aurora kinase. Growth of human tumor xenografts in nude mice was dramatically inhibited after oral administration of MLN8054 in human tumor xenografts. MLN8054 induced mitotic accumulation and apoptosis. Given these findings MLN8054 is currently being explored in the management of patients with platinum-refractory or resistant epithelial, fallopian, or primary peritoneal carcinoma (NCT00853307).

## 11. Hedgehog Pathway Inhibitors

Hedgehog signaling plays a role in many processes such as cell differentiation, growth, and proliferation. This pathway is active during embryonic development and remains active in the adult where it is involved in the maintenance of stem cell populations. 

The Hedgehog family [99] has several proteins which function as signaling molecules. These include Sonic hedgehog (Shh), Indian hedgehog (Ihh), and Desert hedgehog (Dhh). There are two receptors that are involved in the Hedgehog pathway. PATCHED1 is a hedgehog receptor. In the absence of a ligand PATCHED1 inhibits SMOOTHENED, a transmembrane G-coupled protein. However, when the ligand binds PATCHED1, SMOOTHENED suppression is relieved resulting in transcription of the Hedgehog genes. PATCHED1 or SMOOTHENED receptor mutations or overexpression of the Hedgehog ligand leads to uncontrolled cell proliferation.

Bhattacharya et al. [99] studied the role of Hedgehog signaling in ovarian cancer. They utilized a hedgehog pathway blocker and studied the proliferation of ovarian tumors. They noted that PATCHED1 is downregulated in ovarian cancer and that this low level expression of the PATCHED1 contributed to the proliferation of ovarian cancer cells. Chen et al. [100] also examined the expression and the functional role of the hedgehog signal molecules in ovarian cancer. They reported that the hedgehog molecules (Shh, Dhh, Ptch, Smo, and Gli 1 proteins) were increased in malignant disease. Decreased cell proliferation in ovarian carcinoma cell lines was observed with Hedgehog pathway inhibitor- cyclopamine.

Recently reported was the effect of IPI-926 (Infinity Pharmaceuticals, Inc., Cambridge, Mass) a novel inhibitor of the Hedgehog signaling pathway in ovarian cancer grafts. Data revealed that treatment with cyclopamine, the natural product of IPI-926 in animals with primary ovarian cancer grafts, resulted in tumor growth inhibition. This agent is currently being explored as a Phase I study in patients with solid tumors (NCT00761696). 

Currently recruiting is a study of GDC-0449 (Genentech, Inc), a Hedgehog pathway inhibitor, as maintenance therapy in patients with ovarian cancer in a second or third complete remission. GDC-0449 will be evaluated in approximately 100 patients with ovarian cancer in second or third complete remission in a randomized, placebo-controlled, double-blind, multicenter Phase II trial. Patients are randomized in a 1 : 1 ratio to receive either GDC-0449 or a placebo comparator and are stratified based on whether their cancer is in a second or third complete remission. The primary endpoint of the trial is progression-free survival. Secondary outcome measures include overall survival, measurement of Hedgehog ligand expression in archival tissue, and number and attribution of adverse events.

## 12. MTOR Inhibitors

Numerous investigators have reported alterations in PTEN in gynecological malignancies [101]. PTEN is a lipid phosphatase that is associated with cell cycle G1-phase arrest and apoptosis through the PI3K/AKT/mTOR pathway [102]. The mTOR pathway is a central regulator of cell growth, proliferation, and apoptosis. The loss of functional PTEN either through deletion, mutation, or inactivation leads to the constitutive activation of PI3K effectors in the absence of exogenous stimuli. Potential therapies targeting the mTOR pathway include mTOR inhibitors Temsirolimus (CCI-779), everolimus (RAD001), and deforolimus (AP23573).

In ovarian cancer, AKT activity is frequently elevated and is closely associated with the upregulation of mTOR signaling [103]. High levels of AKT activity in vitro result in hypersensitivity to mTOR inhibitors [103]. An in vivo study [104] using xenografts of SKOV-3 cells revealed that RAD001 inhibited tumor growth, angiogenesis, and production of ascites suggesting the potential of mTOR inhibitors in the treatment of women with ovarian cancer.

GOG trial 170I has recently closed a Phase II Evaluation of Temsirolimus (CCI-779, mTOR inhibitor) in the Treatment of Persistent or Recurrent Epithelial Ovarian, Fallopian Tube or Primary Peritoneal Carcinoma. Currently recruiting studies include (NCT00926107), a study of the mTOR inhibitor Temsirolimus (CCI-779) to treat ovarian cancer with Ca125 relapse only, a Phase I study of DOXIL and Temsirolimus in Resistant Solid Malignancies NCT00703170, and a Phase I study of Docetaxel and Temsirolimus in resistant solid malignancies (NCT00703625).

## 13. Conclusion

Multiple attractive targets for the design of targeted therapeutics in ovarian cancer are currently under investigation. Recent studies employing monoclonal antibodies have revealed improvements in time to progression. Studies with tyrosine kinases inhibitors remain in their infancy of development but have provided the basis for continued research. 

Despite these advances there are multiple goals for the future. These include a better understanding of the redundant pathways that exist in cell signaling, creative targeting of horizontal and vertical signaling pathways, identification of other predictive markers to better identify a targeted subpopulation of patients that will respond, and an underlying of the mechanisms of resistance. Achieving these goals will be of paramount importance in the study of targeted therapy in ovarian cancer.

## Figures and Tables

**Table 1:** Current trials in ovarian/fallopian/peritoneal cancer.

Author	N	Prior lines	Platinum S/R*/first line	Regimen**	RR:CR + PR	TTP/PFS median
Burger R et al. [7]	62	1-2	+/+	Single	21 %	PFS 4.7mo
Cannistra et al. [8]	44	2-3	+/+	Single	15.9%	PFS 4.4 mo
Garcia et al. [9]	70	1–3	+/+	Combo	24%	TTP 7.2 mo
Wright et al. [10]	23	2–15	−/+	Combo	35 %	TTP 5.6 mo
Chura et al. [11]	15	5–15	+/+	Combo	43 %	PFS 3.9 mo
Nimeiri et al. [12]	13	1–3	+/+	Combo	15 %	PFS 4.1 mo
Monk et al. [13]	32	2–10	−/+	Single	16 %	PFS 5.5 mo
Simpkins et al. [14]	25	2–12	−/+	Combo	28 %	TTP 9.0 mo
McGonigle et al. [15]	18	0–2	−/+	Combo	22%	PFS 3.8 mo
Azad et al. [17]	13	NR	NR	Combo	46%	NR
Micha et al. [16]	20	0	First line	Combo	80%	NR
Campos/Penson et al. [18, 19]	58	0	First line	Combo	75%	PFS:11mo

*Enrolled patients: platinum sensitive/resistant/first line.

**Single bevacizumab or combination therapy with cytotoxic or other biological agents.

NR: not reported.

**Table 2:** PDGF-targeted therapies in ovarian cancer.

clinical trial.gov ID	Therapeutic regimen	Study PI
NCT00913835	Doxil ± IMC 3G3 in platinum refractory or resistant EOC	W. McGuire
NCT00768144	Sunitinib in refractory/recurrent ovarian, fallopian tube, or peritoneal cancer	S. Campos
NCT00437372	Sunitinib and radiation therapy	A . Dicker
NCT00792545	Dasatinib + bevacizumab in surgically metastatic, or unresectable solid tumors	E. Kohn
NCT00672295	Dasatinib + paclitaxel + carboplatin in ovarian, fallopian tube, and peritoneal cancer	A. Secord
NCT00436215	Sorafenib + bevacizumab in recurrent/refractory ovarian, fallopian tube, or peritoneal cancer	E. Kohn
NCT00526799	Sorafenib + topotecan in platinum resistant EOC	D. Matei
NCT00390611	Paclitaxel + carboplatin ± sorafenib for first-line therapy for EOC	J. Hainsworth
NCT00096200,	Sorafenib + paclitaxel + carboplatin in recurrent platinum-sensitive ovarian, fallopian tube, or peritoneal cancer	V. von Gruenigan
NCT00510653	Gleevac study for patients with ovarian cancer	D. Gershenson
NCT00840450	Gleevac and paclitaxel with recurrent mullerian cancers	F. Muggia

**Table 3:** *α*-folate receptor inhibitors and ovarian cancer.

Clinical trial.gov ID	Therapeutic regimen	Study PI
NCT00722592	Doxil and EC145in platinum resistant EOC	R. Messmann
NCT00738699	MORAb-003 in first platinum resistant or refractory relapsed EOC	D. Chakraborty
NCT00849667	MORAb-003 in platinum sensitive, first relapse EOC	D. Chakraborty

**Table 4:** PARP inhibitors and ovarian cancer.

clinical trial.gov ID	Therapeutic regimen	Study PI
NCT00753545	AZD2281 in platinum sensitive EOC	J. Lederman
NCT00679783	AZD2281 in known BRCA or recurrent EOC	K. Gelman
NCT00749502	MK4827 in BRCA mutant ovarian cancer	
NCT00664781	AG014699 in BRCA mutant ovarian cancer	R. Plummer
NCT00647062	AZD2281 and carboplatin in BRCA mutant EOC	E. Kohn
